# Patterns of pain over time among children with juvenile idiopathic arthritis

**DOI:** 10.1136/archdischild-2017-313337

**Published:** 2017-11-25

**Authors:** Amir Rashid, Lis Cordingley, Roberto Carrasco, Helen E Foster, Eileen M Baildam, Alice Chieng, Joyce E Davidson, Lucy R Wedderburn, Yiannis Ioannou, Flora McErlane, Suzanne M M Verstappen, Kimme L Hyrich, Wendy Thomson

**Affiliations:** 1 Arthritis Research UK Centre for Epidemiology, The University of Manchester, Manchester, UK; 2 NIHR Manchester Musculoskeletal Biomedical Research Unit, Manchester Academic Health Science Centre, Central Manchester University Hospitals NHS Foundation Trust, Manchester, UK; 3 Musculoskeletal Research Group, Institute Cellular Medicine, Newcastle University, Newcastle, UK; 4 Paediatric Rheumatology, Great North Children’s Hospital, Newcastle, UK; 5 Paediatric Rheumatology, Alder Hey Children’s NHS Foundation Trust, Liverpool, UK; 6 Paediatric Rheumatology, Royal Manchester Children’s Hospital, Manchester, UK; 7 Paediatric Rheumatology, Royal Hospital for Children, Glasgow, UK; 8 Paediatric Rheumatology, Royal Hospital for Sick Children, Edinburgh, UK; 9 University College London (UCL) GOS Institute of Child Health, Great Ormond Street Hospital For Children NHS Trust, London, UK; 10 ARUK Centre for Adolescent Rheumatology, University College London, London, UK; 11 The NIHR Biomedical Research Centre, Great Ormond Street Hospital for Children NHS Trust, London, UK; 12 Arthritis Research UK Center for Genetics and Genomics, The University of Manchester, London, UK

**Keywords:** pain, adolescent health, epidemiology, musculo-skeletal, rheumatology

## Abstract

**Objectives:**

Pain is a very common symptom of juvenile idiopathic arthritis (JIA). Disease activity alone cannot explain symptoms of pain in all children, suggesting other factors may be relevant. The objectives of this study were to describe the different patterns of pain experienced over time in children with JIA and to identify predictors of which children are likely to experience ongoing pain.

**Methods:**

This study used longitudinal-data from patients (aged 1–16 years) with new-onset JIA. Baseline and up to 5-year follow-up pain data from the Childhood Arthritis Prospective Study (CAPS) were used. A two-step approach was adopted. First, pain trajectories were modelled using a discrete mixture model. Second, multinomial logistic regression was used to determine the association between variables and trajectories.

**Results:**

Data from 851 individuals were included (4 years, median follow-up). A three-group trajectory model was identified: consistently low pain (n=453), improved pain (n=254) and consistently high pain (n=144). Children with improved pain or consistently high pain differed on average at baseline from consistently low pain. Older age at onset, poor function/disability and longer disease duration at baseline were associated with consistently high pain compared with consistently low pain. Early increases in pain and poor function/disability were also associated with consistently high pain compared with consistently low pain.

**Conclusions:**

This study has identified routinely collected clinical factors, which may indicate those individuals with JIA at risk of poor pain outcomes earlier in disease. Identifying those at highest risk of poor pain outcomes at disease onset may enable targeted pain management strategies to be implemented early in disease thus reducing the risk of poor pain outcomes.

**What is already known on this topic?**

- Juvenile idiopathic arthritis (JIA) is the most common form of inflammatory arthritis in children and young people.
- Pain is one of the most common symptoms reported by children and young people with JIA.
- Pain in JIA is poorly understood and unsatisfactorily managed for children and young people.

**What this study adds?**

- Children with JIA classify into three pain groups: consistently low pain, improved pain and consistently high pain. A clinically significant proportion of patients experience high pain that persists.
- Factors at presentation including age at onset, disease duration, functional disability and pain are identified as associated with consistently high pain over time.
- This study will help identify children at the highest risk of chronic pain, who should be targeted early with multidisciplinary pain management interventions.

## Introduction

Inflammatory arthritis in children is a chronic and often disabling disease. With an annual incidence of approximately 33/100 000 children, juvenile idiopathic arthritis (JIA) represents the most common form of inflammatory arthritis in children.^1^ The disease is heterogeneous,^2^ its course variable with periods of activity and remission^3^ and can severely reduce quality of life.^2 4 5^ Up to a third of young people report active disease progressing into adult life.^6^

Pain is a commonly reported symptom in JIA^7 8^ and a recent study of illness perceptions in adolescents with JIA found that beliefs about their disease activity was influenced by their experience of pain.^9^ A study of individuals with polyarticular JIA found that pain was reported almost daily, with 31% reporting severe pain.^10^ Such levels of pain interfere with physical, educational, emotional and social activity, even when disease activity is controlled.^11 12^ Moreover, a significant number of patients continue to report pain into adulthood.^13^ This may explain the emerging consensus that the management of JIA needs to address more than active inflammation^14^ to minimise the long-term impact of the disease.

Active disease, although influential,^15–17^ accounts for small amounts of variance in pain in patients with JIA.^15 18^ Considerable variance in pain remains unaccounted for and increases as the disease progresses into adulthood.^4^ We know little about the different possible patterns of pain in JIA over time or which factors predict them. Previous studies were limited in their ability to provide greater understanding due to the nature and extent of the data collected, being either cross-sectional data or longitudinal data with small samples.^19 20^ They also focused on examining relationships between variables (variable-centred analyses). The aims of this analysis are to identify and group children with similar patterns of pain over time from baseline (person-centred analysis), and thereby predict patients with JIA at risk of poor pain outcomes.^21^

## Method

### Study population

The participants in this study were recruited to the Childhood Arthritis Prospective Study (CAPS). This longitudinal prospective inception cohort study of individuals with new-onset inflammatory arthritis, primarily JIA, was established in 2001, in the UK. Methodological details of this study have been published elsewhere.^22^ Patients are recruited into CAPS at first presentation to paediatric rheumatology (baseline). Written informed consent was provided by parent(s)/guardian(s) and patients (aged 16 years and older) in accordance with the Declaration of Helsinki.

### Data collection

Data items used in the present analysis included the 10 cm pain visual analogue scale (VAS) (580 proxy reports and 271 child reports), active joint count (AJC) from 71 joints, 10 cm physician’s global assessment (PGA) VAS, 10 cm patient/proxy general evaluation (PGE) VAS, Child Health Assessment Questionnaire (CHAQ: functional disability) scored 0–3, Moods and Feelings Questionnaire (MFQ) scored 0–66 (self-report) and 0–68 (proxy-report) and the physician-assigned International League Against Rheumatism (ILAR) subtype. In addition, this analysis also included medication taken in the first year (eg, disease-modifying antirheumatic drugs (DMARDs), biologics and steroids). Data included in the analysis were collected at baseline (presentation), 6 months, 1 year and then annually up to 5 years. The data were extracted from hospital records and questionnaires.

### Analysis

The primary outcome measure was the 10 cm pain VAS scores. Only children with a JIA subtype recorded at year 1 and pain scores recorded at baseline plus at least one additional follow-up pain score (up to 5 years postbaseline) were included in the analysis. A two-stage approach to this analysis was used.^23^ First, pain trajectories of patients were modelled using a form of discrete mixture-modelling called group-based trajectory analysis (GBTA).^24^ GBTA assigns each individual to a trajectory group, based on Bayesian posterior probabilities for group membership.^21^ Group-based trajectories were modelled in groups ranging from two to six trajectories. The optimal number of possible trajectories was determined by statistical fit (including Bayesian information criterion and model parsimony^21^) and the clinical relevance determined by the research team.

In the second step, multivariable multinomial logistic regression models were used to determine the association between possible predictors and membership in the trajectory groups.^23 25^ Predictors were chosen based on their salience to JIA and paediatric pain. They included baseline measures of age at onset, gender, pain, AJC, PGA, PGE, CHAQ, MFQ and disease duration (at baseline), plus early change measures of pain, AJC, PGA, PGE, CHAQ and medication. The predictors were entered into the model simultaneously. To deal with missing covariate data (table 1), multiple imputation (chained equations^26^) was used, with 50 datasets generated using the rule of thumb that the minimum number of imputations equated to the percentage of incomplete cases.^26^ Data analysis was conducted using STATA V.12.0, and user-written STATA modules *Kwallis2* and *TRAJ*.^27^

**Table 1:** Characteristics of the study population as a whole (n=851) and stratified by pain trajectory

Variable	Available data, n (% missing)	All patients	Consistently low pain, n=453 (50.2%)	Improved pain, n=254 (31.9%)	Consistently high pain, n=144 (17.9%)
Baseline					
Age, years*	847 (0.5)	7.6 (3.6–11.8)	6.3 (2.9–10.9)†‡	8.6 (3.7–12.3)†§	10.8 (6.5–13.2)‡§
Age at onset, years*	843 (0.9)	6.6 (2.7–10.8)	5.6 (2.4–9.9)†‡	7.0 (3.0–11.1)†§	9.3 (4.5–11.8)‡§
Gender n (%¶)	851 (0.0)				
Female		562 (66.0)	288 (63.6)**	184 (72.4)**	90 (62.5)**
Male		289 (34.0)	165 (36.4)	70 (27.6)	54 (37.5)
Disease duration, months*	839 (1.4)	5.3 (2.7–10.7)	4.7 (2.3–8.5)†‡	5.8 (2.8–12.8) †§	6.9 (3.7–17.6)‡§
Disease duration from referral, weeks*	793 (6.8)	4 (1.3–7.7)	4 (1.4–7.7)	3.4 (1–7.1)	4.6 (1.3–8.1)
PGA, cm*	600 (29.5)	3 (1.8, 5.4)	2.6 (1.4, 4.8)†‡	3.5 (2, 5.7)†	3.3 (2.1, 5.8)‡
AJC*	780 (8.3)	2 (1–6)	2 (1, 5)†‡	3 (1, 8)†	3 (1, 8)‡
PGE, cm*	834 (2.0)	2.1 (0.5, 5)	0.8 (0.1, 2.4)†‡	4.6 (2.2, 6.2)†	4.1 (2, 6.35)‡
Pain, cm*	851 (0.0)	3 (0.8, 5.7)	1 (0.2, 2.5)†‡	6 (4.5, 7.4)†§	5 (3, 6.9)‡§
CHAQ*	849 (0.2)	0.6 (0.1–1.4)	0.3 (0–0.9)†‡	1.1 (0.6–1.8)†	1.1 (0.6–1.8)‡
MFQ*	312 (63.3)	13 (6, 23)	9 (4, 16)	17 (9, 27)	21 (13, 27)
Early change					
DMARDs 1st year n (%¶)	851 (0.0)				
No		434 (51)	274 (60.5)**	101 (39.8)**	59 (41.0)**
Yes		417 (49)	179 (39.5)	153 (60.2)	85 (59.0)
Number of DMARDs 1st year n (%¶)	851 (0.0)				
1		381 (91.4)	169 (94.4)**	141 (92.2)**	71 (83.5)**
2		31 (7.4)	8 (4.5)	10 (6.5)	13 (15.3)
3		5 (1.2)	2 (1.1)	2 (1.3)	1 (1.2)
Biologics 1st year n (%¶)	851 (0.0)				
No		769 (90.4)	430 (94.9)**	222 (87.4)**	117 (81.3)**
Yes		82 (9.6)	23 (5.1)	32 (12.6)	27 (18.7)
Number of biologics 1st year n (%¶)	851 (0.0)				
1		75 (91.5)	22 (95.6)	28 (87.5)	25 (92.6)
2		6 (7.3)	1 (4.4)	3 (9.4)	2 (7.4)
3		1 (1.2)	0 (0)	1 (3.1)	0 (0.0)
All steroids 1st year n (%¶)	851 (0.0)				
No		207 (24.3)	118 (26.1)	56 (22.1)	33 (22.9)
Yes		644 (75.7)	335 (73.9)	198 (77.9)	111 (77.1)
Change in AJC 6 months, cm*††	541 (36.4)	−1 (−4, 0)	−1 (−3, 0)	−1 (−5, 0)	−1 (−4, 0)
Change in PGA 6 months, cm*††	390 (54.2)	−1.9 (−3.9, –0.5)	−1.9 (−4.2, –0.7)	−1.9 (−3.4, –0.3)	−1.8 (−3.9, –0.2)
Change in PGE 6 months, cm*††	500 (41.2)	−0.5 (–2.4, 0.3)	−0.3 (–0.2, 0.1)	−1.2 (–3.5, 0.6)§	−0.1 (–2, 1.5)^§^
Change in CHAQ 1 year*††	702 (17.7)	−0.1 (–0.6, 0)	−0.1 (–0.6, 0)†‡	−0.4 (–1, 0)†§	0 (–0.5, 0.4)‡§
Change in pain 1 year, cm*††	691 (18.8)	−0.7 (–3.2, 0.2)	−0.4 (–1.9, 0)†‡	−3.2 (–5.8, –0.3)†§	0.5 (–1.7, 2.6)‡§

All values are median (IQR) unless stated.

*Multiple comparisons of significant differences between trajectory groups using an adjusted P≤0.0083 from Kruskal-Wallis tests. n, number of children.

†Improved pain versus consistently low pain.

‡Consistently high pain versus consistently low pain.

§Consistently high pain versus improved pain.

¶Column.; PGA, Physician’s global assessment PGE, Parent’s/patient’s general evaluation of wellbeingwell-being VAS, visual analogue scale; CHAQ, Childhood Health Assessment Questionnaire.

**P≤0.05 from χ^2^ indicates a significant difference in proportions between the three trajectory groups.

††Minus denotes a decrease in the covariate.

AJC, active joint count; CHAQ, Childhood Health Assessment Questionnaire; n, number of children; nr, not reported; ns, non-significant; PGA, Physician’s Global Assessment; PGE, parent’s/patient’s general evaluation of well-being; VAS, visual analogue scale.

## Results

### Study population

In total, 851 patients were included in this analysis. The median age at diagnosis was 7.6 years (IQR 3.6, 11.8), the median age at disease onset was 6.6 years (IQR 2.7, 10.8) and median disease duration at presentation was 5.3 months (IQR 2.7, 10.7) (table 1); 66% were female. The most common ILAR subtype recorded at 1 year was persistent oligoarthritis (n=265, 42.7%) (table 2). There were negligible differences in characteristics between those who were included and excluded from this analysis (see online supplementary table 1).

10.1136/archdischild-2017-313337.supp1 Supplementary file 1

**Table 2:** Distribution of year 1 physician-assigned ILAR subtypes stratified by pain trajectory, n=851

Variable	All patients n (%)	Consistently low pain, n=453 (50.2%)	Improved pain, n=254 (31.9%)	Consistently high pain, n=144 (17.9%)	P value
Subtype at 1 year	620	317 (51.1)	198 (31.9)	105 (16.9)	0.001^*^
Systemic	39 (6.3)	16 (5.1)† (41.0)	17 (8.6)† (43.6)	6 (5.7)† (15.4)	
Oligoarthritis (persistent)	265 (42.7)	165 (52.1)† (62.3)	64 (32.3)† (24.2)	36 (34.3)† (13.6)	
Oligoarthritis (extended)	33 (5.3)	11 (3.5)† (33.3)	14 (7.1)† (42.4)	8 (7.6)† (24.2)	
Polyarthritis (RF–)	158 (25.5)	75 (23.7)† (47.5)	59 (29.8)† (37.3)	24 (22.9)† (15.2)	
Polyarthritis (RF+)	23 (3.7)	4 (1.3)† (17.4)	13 (6.6)† (56.5)	6 (5.7)† (26.1)	
Enthesitis-related arthritis	33 (5.3)	13 (4.1)† (39.4)	10 (5.1)† (30.3)	10 (9.5)† (30.3)	
Psoriatic arthritis	52 (8.4)	22 (6.9)† (42.3)	16 (8.1)† (30.8)	14 (13.3)† (26.9)	
Undifferentiated	17 (2.7)	11 (3.5)† (64.7)	5 (2.5)† (29.4)	1 (1.0)† (5.9)	

All values are n (column %) unless stated.

*P≤0.05 from χ^2^.

† % is for row.

ILAR, International League Against Rheumatism; RF, rheumatoid factor.

### Trajectory model

**Figure 1:**
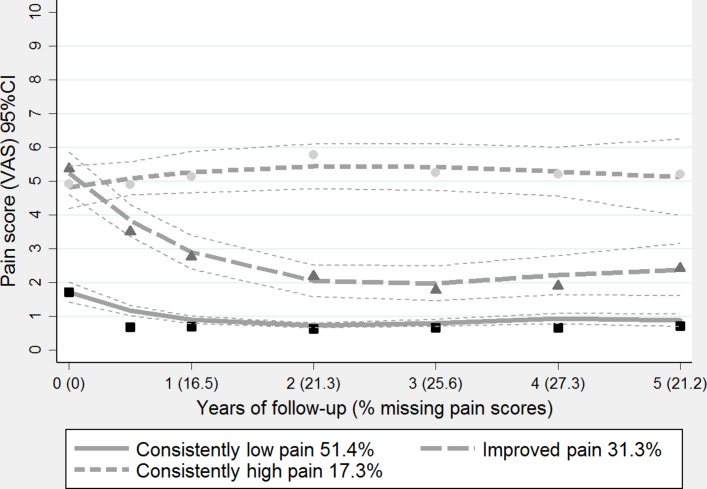
Pain trajectories from baseline to 5-year follow-up. VAS, visual analogue scale.

The three-group model of pain (figure 1) was selected as the most clinically and statistically satisfactory (see online supplementary table 2). Over 78% of children had a >0.7 probability of membership in their assigned trajectory groups. The averaged posterior probabilities of group membership for each of the subgroups were all high (>0.8) with the smallest trajectory group representing 17% of the study population.

The largest group in the three-trajectory model was ‘consistently low’ pain, which accounted for half of the participants in this analysis (figure 1). This consisted of a horizontal trajectory undulating around the 1 cm point of the pain VAS (starting at 1.8 cm) throughout the 5-year period. The second group was ‘improved pain’, which accounted for almost a third of participants. This trajectory (starting at 5.5 cm) illustrated dramatic improvement in pain in the first year of follow-up, leading to a plateau for the subsequent 4 years. The third trajectory (starting at 4.9 cm) was ‘consistently high’ pain, which again was broadly horizontal, oscillating around the 5 cm point of the pain VAS throughout the 5-year follow-up.

### Trajectory group characteristics

Subgroup analysis illustrated clear differences on average (median) between low persistent pain and the other pain trajectories across the majority of covariates (table 1). On average (median), older age at onset, older age at baseline, baseline pain, longer disease duration at baseline and early changes in PGE, pain and CHAQ, distinguished between trajectories with consistently high pain and improved pain (table 1).

Overall, 17% of the study cohort was in the consistently high pain group, this increased to approximately 25% for extended oligoarthritis (n=8, 24.2%), polyarthritis RF+ (n=6, 26.1%) and psoriatic arthritis (n=14, 26.9%) patients, and over 30% for children with enthesitis-related arthritis (n=10, 30.3%). While 42.7% of the study cohorts were classified with persistent oligoarthritis, this increased to over half of the children in the consistently low pain group and down to approximately one-third of the children in each of the other two groups.

### Multinomial logistic regression

Variables associated with improved pain when compared with consistently low pain included being female, and longer disease duration, higher PGE and higher pain at baseline (table 3). In addition, associated early change factors included increased PGE in first 6 months, and increased CHAQ and pain in the first year (table 3). When comparing consistently high pain with consistently low pain, baseline factors of older age at onset, longer disease duration, higher PGE, higher CHAQ and higher pain were associated with consistently high pain membership, along with early change factors including treatment with biologics in the first year, increased PGE in the first 6 months and increased CHAQ and pain in the first year (table 3). Finally, when comparing consistently high pain with improved pain, the baseline factors of older age at onset and higher CHAQ were associated with consistently high pain, along with the early change factors of increased PGE in the first 6 months, and increased CHAQ and pain in the first year (table 3).

**Table 3:** Association between variables and pain trajectories from a multivariable multinomial logistic regression using 50 multiple imputed datasets (n=851)

Variable	Relative risk ratio (95% CI)		
	Improved versus Consistently low	Consistently high versus consistently low	Consistently high versus improved
Baseline			
Gender (base: female)			
Male	0.5 (0.3 to 0.9)*	0.7 (0.4 to 1.4)	1.5 (0.9 to 2.5)
Age at onset, per year	1.1 (0.99 to 1.1)	1.1 (1.1 to 1.2)*	1.1 (1.0 to 1.2)*
PGA, per cm	1.1 (0.9 to 1.3)	1.1 (0.9 to 1.3)	1.0 (0.8 to 1.2)
Disease duration, per month	1.02 (1.0 to 1.04)*	1.03 (1.0 to 1.1)*	1.0 (0.99 to 1.0)
AJC, per joint	0.9 (0.8 to 1.1)	0.95 (0.8 to 1.1)	1.0 (0.9 to 1.1)
PGE, per cm	1.3 (1.0 to 1.5)*	1.4 (1.1 to 1.7)*	1.1 (0.9 to 1.3)
CHAQ, per 0.125	1.1 (0.96 to 1.1)	1.2 (1.0 to 1.3)*	1.1 (1.0 to 1.2)*
Pain, per cm	3.2 (2.5 to 4.1)*	3.1 (2.4 to 4.0)*	1.0 (0.8 to 1.1)
MFQ, per 5%	1.3 (0.7 to 2.5)	1.3 (0.6 to 2.8)	1.0 (0.6 to 1.7)
Early change			
DMARDs in 1st year (base: no)			
Yes	1.2 (0.7 to 2.1)	0.8 (0.4 to 1.5)	0.6 (0.4 to 1.1)*
Biologics in 1st year (base: no)			
Yes	2.3 (0.8 to 6.1)	3.0 (1.0 to 8.8)*	1.3 (0.7 to 2.6)
Steroids in 1st year (base: no)			
Yes	1.0 (0.5 to 1.9)	1.4 (0.7 to 3.1)	1.4 (0.8 to 2.6)
Change in AJC in 1st 6 months, per joint increase	0.9 (0.8 to 1.1)	1.0 (0.8 to 1.1)	1.0 (0.9 to 1.1)
Change in PGA in 1st 6 months, per cm increase	1.2 (0.9 to 1.4)	1.1 (0.9 to 1.4)	1.0 (0.8 to 1.2)
Change in PGE in 1st 6 months, per cm increase	1.3 (1.1 to 1.5)*	1.4 (1.2 to 1.7)*	1.1 (1.0 to 1.3)*
Change in CHAQ in 1st year, per 0.125 increase	1.1 (1.01 to 1.2)*	1.2 (1.1 to 1.3)*	1.1 (1.0 to 1.1)*
Change pain in 1st year, per cm increase	1.4 (1.2 to 1.7)*	1.7 (1.4 to 2.1)*	1.2 (1.1 to 1.3)*

*P≤0.05.

CHAQ, Childhood Health Assessment Questionnaire; DMARDs, disease-modifying antirheumatic drugs; MFQ, Moods and Feelings Questionnaire; PGA, Physician’s Global Assessment; PGE, parent’s/patient’s general evaluation.

## Discussion

Using GBTA, relatively novel in paediatric research, three distinct trajectories of pain in children and young people with JIA have been identified. Although a majority of children report low pain or pain which improves quickly during the first year following diagnosis, almost a fifth of children reported consistently high levels of pain over time. Pain at presentation is by far the strongest, but not only predictor of consistently high pain, consistent with previous research.^20 28^ Neither AJC nor PGA at presentation, nor early changes in AJC or PGA, were found to predict membership in either of the three trajectory groups. Consequently, a management approach focused primarily on disease activity may not necessarily lead to improvements in long-term pain. Children with consistently high pain were more likely to have been prescribed DMARDs and biologics compared with those in the other pain trajectories within the first 5 year of follow-up (see online supplementary table 3). This is consistent with findings reported by others that a significant proportion of patients with JIA treated with biologics including anti-TNF treatments still experienced pain^12 29^ with 19% of patients reporting severe pain.^30^ Similarly, this study found that being treated with biologics within the first year almost trebled a child’s likelihood of consistently high pain membership. Clinicians must address disease activity in their treatment decisions but must also address factors such as pain when considering the best management approach for JIA.^31^

A notable finding was that baseline measures of disease activity such as the AJC and PGA, did not predict children’s membership in any pain trajectory group. In contrast, baseline evaluations of patients or proxy (CHAQ, PGE and pain) scores did inform membership in the pain trajectories, as did early changes in those measures. Baseline functional disability (CHAQ) was a strong predictor of membership of the consistently high pain trajectory. Packham and Hall in their cross-sectional study found that functional disability contributed 18% of total variance explained in pain.^4^ Interestingly, an analysis of the CAPS cohort after a 1-year prospective follow-up found baseline pain to be a predictor of moderate-to-severe functional disability.^32^ These findings demonstrate the complex relationships between pain and functional disability, with experiences of one likely to contribute to the self-report of the other.

Age at onset also appears to have a relationship with trajectory group membership. Patients with consistently low pain were younger on average, those with improved pain were more commonly in the middle childhood age group at onset and those with consistently high pain were, on average, approaching early adolescence.^14^ Malleson *et al* 16 and Weiss *et al* found that older age was predictive of pain in patients with JIA.^14^ Patients who are older at onset may find it more difficult to adjust with the changes to their functioning than younger children^33^ and perceive the long-term consequences of JIA as more significant.^34^ Moreover, they may have to relearn and adjust to new ways of doing things rather than learning to do things for the first time as younger-onset children.^35^ Additionally, the psychological, social and emotional pressures experienced by those whose disease starts in adolescence such as conforming to peer-group expectations, increasing independence and burgeoning identity may magnify their experience of pain and functional disability when compared with those whose disease starts when they are younger.^35^

Disease duration at baseline was also associated with consistently high pain. One explanation for this may be due to the greater potential damage caused by JIA prior to its remission in consistently high pain owing to potential delays in appropriate treatment^22^ (with 21% of patients in this study waiting ≥1 year prior to presentation). Fantini *et al* found attaining remission decreases in proportion to delay in presenting to paediatric rheumatology.^3^ Oen *et al* found that time between onset and diagnosis was a prospective predictor of functional disability at follow-up.^5^ Parent’s unfamiliarity with the urgency of symptoms may delay seeking medical advice^22^; complicated by variables such as socioeconomic status.^36^ Therefore, their disease may go untreated for longer allowing functional disability to increase,^22^ potentially demonstrating why poor functional disability predicted consistently high pain. This may also lead to a greater probability of enhanced sensitivity to all pain-inducing stimuli beyond that induced by affected joints.^7^ This amplification in pain may persist beyond the resolution of inflammation leading to peripheral and central sensitisation,^7^ and lowered pain thresholds, pain tolerances^37^ and high chronic pain, as found in those in consistently high pain.

Contrary to previous research, depression was not found to predict any pain trajectory.^28 38^ In this study, depression was correlated positively with pain (see online supplementary table 4). An explanation for the findings may be that PGE, which is a measure of well-being, occupies similar variance associated with depression, thereby reducing its uniquely associated contribution. Higher PGE scores were found to predict consistently high pain. Patients and/or their parents more readily view pain as an indicator of arthritis^9 39^ than do clinicians. Therefore, PGE scores would provide a more accurate indicator of pain experiences over time than PGA, as it takes into account pain experience.

The findings from this study have a number of implications for pain management in children presenting for the first time to paediatric rheumatology. Alongside objective markers of disease activity, this study suggests that clinicians should ensure that pain is also addressed, particularly among those presenting with higher levels of pain and PGE, poor functional ability, older onset and/or longer disease duration. Importantly, early increases in PGE, poor functional ability and pain are also independently associated with consistently high pain, indicating that delays in pain management may only serve to increase the risk of a child developing consistently high pain. Hence, pain management should be prioritised early in children with JIA. Furthermore, medications such as DMARDs, biologics and steroids, which are used to reduce disease activity, may be less efficacious in reducing pain in some patients. Therefore, an integrated management approach in which pain is assessed and managed alongside disease activity is needed to reduce the prospect of poor long-term pain outcomes.

### Limitations

These findings must be viewed in the context of some limitations. The pain, PGE, CHAQ and MFQ score was reported by a parent for children under the age of 11 and were self-reported by children aged ≥11 years. The potential limitations of such strategies of data collection have been identified previously.^40^ However, this strategy reflects the routine collection of data in the UK paediatric rheumatology. Therefore, the baseline covariates in this study correspond with data that were available to paediatric rheumatologists. Furthermore, information on pain management was not examined, which may highlight that if interventions were used they were not adequate in reducing pain in at least a fifth of the cohort.

## Conclusion

This study has used clinical data which are routinely collected from patients with JIA to classify pain trajectories into three categories. Factors which contributed to pain patterns included disease features (eg, duration, age at onset, etc), which would enable children with JIA at an increased risk of consistently high pain over time to be identified early in disease. It is important for children with JIA that integrated pain management strategies are used in addition to pharmacological therapy aimed to control inflammation, and targeted at those most at risk, as soon as possible after diagnosis to improve important long-term pain outcomes.
