# Supplementary material for: Patterns of pain over time among children with juvenile idiopathic arthritis
Source: Arch Dis Child. 2017 Nov 25;103(5):437–43. doi: 10.1136/archdischild-2017-313337 (PMC5916104; doi:10.1136/archdischild-2017-313337)
Supplement: Supplementary file 1 [file archdischild-2017-313337supp001.docx]

**Supplementary Data, Rashid, Patterns of Pain over time among children with Juvenile Idiopathic Arthritis**

**Supplementary Table 1. Comparison of characteristics between those selected for this analysis and those not selected, from the CAPS cohort (excluding missing values due to not reaching follow up visit)**

|  | **Year 0.5** | | | **Year 1** | | | **Year 2** | | | **Year 3** | | | **Year 4** | | | **Year 5** | | | |
| --- | --- | --- | --- | --- | --- | --- | --- | --- | --- | --- | --- | --- | --- | --- | --- | --- | --- | --- | --- |
| **Variable** | **Not selected^#^ (n=411)** | **Selected in study^b^ (n=674)** | **p-value** | **Not selected^a^ (n=500)** | **Selected in study^b^ (n=820)** | **P-value** | **Not selected^a^ (n=406)** | **Selected in study^b^ (n=737)** | **P-value** | **Not selected^a^ (n=334)** | **Selected in study^b^ (n=609)** | **P-value** | **Not selected^a^ (n=261)** | **Selected in study^b^ (n=516)** | **P-value** | **Not selected^a^ (n=206)** | | **Selected in study^b^ (n=419)** | **P-value** |
| **Age in years** | 8.3 (3.5,12.1) | 7.6 (3.6,11.6) | 0.4 | 7.8 (3.5,12.2) | 7.6 (3.5,11.8) | 0.5 | 7.5 (3.5,11.9) | 7.4 (3.3,11.5) | 0.6 | 7.3 (3.3,11.4) | 7.3 (3.3,11.3) | 0.9 | 8.4 (4.5,11.3) | 6.5 (2.8,10.7) | 0.001 | 7.3 (3.7,10.8) | 6.3 (2.7,10.2) | | 0.03 |
| **Age at onset in years** | 6.8 (2.7,10.8) | 6.6 (2.7,10.6) | 0.7 | 6.7 (2.5,10.8) | 6.8 (2.7,10.7) | 0.9 | 6.4 (2.6,10.6) | 6.4 (2.5,10.6) | 0.9 | 6.2 (2.5,10.4) | 6.3 (2.5,10.4) | 0.7 | 7.1 (3.4,10.6) | 5.5  (1.2,9.5) | 0.0004 | 6.1  (2.9,9.9) | 5.3  (2.1,9.1) | | 0.05 |
| **Gender n(%)**  **Female**  **Male** | 243(59.1)  168(40.9) | 438 (65.0)  236 (35.0) | 0.1^c^ | 295(59.2)  203(40.8) | 544(66.3)  276(33.7) | 0.01^c^ | 243 (59.9)  163 (40.1) | 489 (66.4)  248 (33.6) | 0.03^c^ | 196 (58.9)  137 (41.1) | 407 (66.8)  202 (33.2) | 0.02^c^ | 159 (63.4)  92 (36.7) | 348 (66.2)  178 (33.8) | 0.4^c^ | 124 (72.1)  48 (27.9) | 288 (64.1)  161 (35.9) | | 0.1^c^ |
| **Disease duration in months** | 5.5 (3.1,12.2) | 5.5 (2.8,10.7) | 0.2 | 5.5 (3.0,12.4) | 5.4 (2.8,10.9) | 0.1 | 5.4 (3.0,12.1) | 5.4 (2.8,10.3) | 0.3 | 5.3 (2.9,11.2) | 5.4 (2.8,10.6) | 0.5 | 5.5  (1.0,9.8) | 5.5 (2.8,10.6) | 0.4 | 5.6 (3.0,10.7) | 4.9 (2.7,10.9) | | 0.1 |
| **Disease duration from referral (weeks)** | 4.3 (1.6,8.9) | 3.9 (1.3,7.6) | 0.05 | 4.1 (1.6,8.3) | 3.9 (1.3,7.7) | 0.2 | 4.3 (1.7,8.9) | 3.9  (1.3,7.6) | 0.1 | 4.1  (1.5,8.1) | 3.9  (1.3,7.7) | 0.3 | 4.1  (1.3,7.6) | 3.6  (1.3,7.4) | 0.5 | 3.9 (1.3,13.1) | 3.6  (1.3,7.3) | | 0.9 |
| **PGA** | 28 (14,47) | 30 (18,54) | 0.01 | 28 (14,47) | 30  (18,54) | 0.03 | 29  (15,47) | 30  (17,54) | 0.1 | 29  (14,49) | 30  (18,54) | 0.04 | 29  (15,50) | 30  (17,53) | 0.5 | 28  (17,50) | 31  (18,54) | | 0.4 |
| **Active Joint Count** | 2  (1,5) | 2  (1,5) | 0.3 | 2  (1,5) | 2  (1,6) | 0.7 | 2  (1,5) | 2  (1,6) | 0.9 | 2  (1,5) | 2  (1,6) | 0.5 | 2  (1,5) | 2  (1,6) | 0.3 | 2  (1,5) | 2  (1,6) | | 0.7 |
| **PGE** | 44 (12,68) | 21 (5,50) | 0.01 | 43  (15,66) | 21  (5,50) | 0.001 | 40.5 (20,66) | 21  (5,50) | 0.01 | 34  (24,56) | 21  (5,50) | 0.01 | 30 (7,50) | 20 (5,46) | 0.01 | 29  (6,54) | 19  (4,46) | | 0.03 |
| **Pain VAS** | 43.5 (20,69.5) | 30 (8,58) | 0.03 | 44  (19,69) | 30  (8,58) | 0.01 | 43.5 (20,69) | 30  (8,57) | 0.03 | 40  (26,75) | 30  (8,58) | 0.01 | 33.5  (10,60) | 28  (7,57) | 0.1 | 39.5 (12.5,62) | 29.5  (7,54) | | 0.1 |
| **CHAQ score** | 0.9 (0.1,1.5) | 0.6 (0.1,1.4) | 0.5 | 0.8 (0.14,1.5) | 0.6 (0.13,1.38) | 0.3 | 0.8 (0.1,1.5) | 0.8  (0.1,1.4) | 0.3 | 0.8  (0.3,1.6) | 0.8  (0.1,1.4) | 0.5 | 1  (0.3,1.5) | 0.6  (0.1,1.3) | 0.02 | 1  (0.4,1.5) | 0.6  (0.1,1.4) | | 0.01 |
| All values are median (IQR) unless stated. ^a^refers to participants whom have provided data at this time point but whom have not provided a baseline pain score and at least one follow-up pain score. ^b^ refers to participants whom have provided data at this time point and whom have provided a baseline pain score and at least one follow-up pain score. P-value of ≤ 0.0056 from kruskal-wallis tests corrected for multiple testing using Bonferroni method, unless otherwise stated. ^c^p-value from chi-square. PGA, Physician’s global assessment; PGE, Parent’s global evaluation of wellbeing; VAS, visual analogue scale; CHAQ, Childhood Health Assessment Questionnaire. | | | | | | | | | | | | | | | | | | | |

**Supplementary Table 2. Comparison between different group numbers of trajectory groups , n=851**

| **No. of groups** | **BIC** | **AIC** | **Log-likelihood (L)** | **% children with ≥0.7 Posterior Probabilities in model** | **Averaged Posterior Probabilities of group membership** | **(%) per group** |
| --- | --- | --- | --- | --- | --- | --- |
| 2 | -14785.28 | -14761.62 | -14751.62 | 89.8 | Group 1: 0.99  Group 2: 0.97 | Group 1 (60.9)  Group 2 (39.1) |
| 3 | -14728.62 | -14693.12 | -14678.12 | 78.5 | Group 1: 0.96  Group 2: 0.81  Group 3: 0.90 | Group 1 (50.2)  Group 2 (31.9)  Group 3 (17.9) |
| 4 | -14725.09 | -14677.76 | -14657.76 | 64.0 | Group 1: 0.67  Group 2: 0.79  Group 3: 0.79  Group 4: 0.90 | Group 1 (13.5)  Group 2 (41.6)  Group 3 (26.5)  Group 4 (18.4) |
| 5 | -14720.43 | -14661.26 | 14636.26 | 61.8 | Group 1: 0.67  Group 2: 0.79  Group 3: 0.74  Group 4: 0.80  Group 5: 0.85 | Group 1 (11.4)  Group 2 (41.5)  Group 3 (10.4)  Group 4 (26.1)  Group 5 (10.7) |
| 6 | -14712.87 | -14641.87 | -14611.87 | 56.0 | Group 1: 0.70  Group 2: 0.68  Group 3: 0.83  Group 4: 0.76  Group 5: 0.78  Group 6: 0.83 | Group 1 (8.5)  Group 2 (5.1)  Group 3 (41.6)  Group 4 (10.7)  Group 5 (23.9)  Group 6 (10.2) |
| BIC, Bayesian Information Criterion; AIC, Akaike Information Criterion; L, Log-likelihood; | | | | | | |

**Treatment**

In five years of follow-up, those with consistently-low pain were less likely to be treated with DMARDS and biologics compared to other pain-trajectories (supplementary table 2). Conversely, individuals with consistently-high pain were more likely to have been treated with DMARDS and biologics compared to other pain-trajectories, which appeared to be independent from ILAR subtype (supplementary tables 3 & 4). There were no differences between pain-trajectories in the proportions receiving steroid treatment (Supplementary table 2)

**Supplementary Table 3. Treatment characteristics by trajectory, n=851**

| **Variable** | **All patients** | **Consistently-low pain, n=453 (50.2%)** | **Improved-pain, n=254 (31.9%)** | **Consistently-high pain, n=144 (17.9%)** | **P value** |
| --- | --- | --- | --- | --- | --- |
| Any DMARDs in the 1^st^ 5 years  No  Yes | 364 (42.8)  487 (57.2) | 243 (53.6)  210 (46.4) | 80 (31.5)  174 (68.5) | 41 (28.5)  103 (71.5) | 0.001**^a^** |
| Any Biologics in the 1^st^ 5 years  No  Yes | 648 (86.2)  203 (23.9) | 387 (85.5)  66 (14.6) | 173 (68.1)  81 (31.9) | 88 (61.1)  56 (38.9) | 0.001**^a^** |
| Any steroids in 1^st^ 5 years  No  Yes | 171 (20.1)  680 (79.9) | 101 (22.3)  352 (77.7) | 42 (16.5)  212 (83.5) | 28 (19.4)  116 (80.6) | 0.182**^a^** |
| All values are n(%) unless stated. **^a^**p-value from chi-square. | | | | | |

**Supplementary Table 4. Correlations of covariates using a 50 multiply imputed datasets**

| **Variable** | **Gender** | **Age at onset** | **Disease duration at baseline** | **AJC at baseline** | **PGA at baseline** | **PGE at baseline** | **CHAQ at baseline** | **Pain at baseline** | **MFQ at baseline** | **DMARDs in 1^st^ year** | **Biologics in 1^st^ year** | **Steroids in 1^st^ year** | **Change AJC in 1^st^ year** | **Change PGA in 1^st^ year** | **Change PGE in 1^st^ year** | **Change CHAQ in 1^st^ year** | **Change Pain in 1^st^ year** |
| --- | --- | --- | --- | --- | --- | --- | --- | --- | --- | --- | --- | --- | --- | --- | --- | --- | --- |
| **Baseline** |  |  |  |  |  |  |  |  |  |  |  |  |  |  |  |  |  |
| Gender | 1 |  |  |  |  |  |  |  |  |  |  |  |  |  |  |  |  |
| Age at onset, years | 0.171*** | 1 |  |  |  |  |  |  |  |  |  |  |  |  |  |  |  |
| Disease duration | 0.032 | 0.050 | 1 |  |  |  |  |  |  |  |  |  |  |  |  |  |  |
| AJC in baseline | -0.01** | 0.085* | 0.110** | 1 |  |  |  |  |  |  |  |  |  |  |  |  |  |
| PGA at baseline | -0.059 | 0.030 | 0.031 | 0.425*** | 1 |  |  |  |  |  |  |  |  |  |  |  |  |
| PGE in baseline | -0.006 | 0.102* | 0.083 | 0.189*** | 0.212*** | 1 |  |  |  |  |  |  |  |  |  |  |  |
| CHAQ in baseline | -0.099** | 0.034 | 0.049 | 0.353*** | 0.314*** | 0.597*** | 1 |  |  |  |  |  |  |  |  |  |  |
| Pain at baseline | -0.033 | 0.096** | 0.080* | 0.206*** | 0.240*** | 0.513***- | 0.604*** | 1 |  |  |  |  |  |  |  |  |  |
| MFQ at baseline | -0.056 | 0.082 | -0.038 | 0.218*** | 0.179* | 0.365*** | 0.465*** | 0.338*** | 1 |  |  |  |  |  |  |  |  |
| **Early change** |  |  |  |  |  |  |  |  |  |  |  |  |  |  |  |  |  |
| DMARDs in 1^st^ year | -0.082* | 0.052 | 0.056 | 0.357*** | 0.318*** | 0.169*** | 0.318*** | 0.218*** | 0.171*** | 1 |  |  |  |  |  |  |  |
| Biologics in 1^st^ year | -0.024 | 0.098*** | 0.088* | 0.265*** | 0.226*** | 0.131*** | 0.206*** | 0.119*** | 0.141*** | 0.309*** | 1 |  |  |  |  |  |  |
| Steroids in 1^st^ year | 0.062 | -0.136*** | 0.023 | 0.054 | 0.154*** | -0.008 | 0.062 | 0.057 | 0.033 | 0.265*** | 0.120*** | 1 |  |  |  |  |  |
| Change AJC 1^st^ 6 months | 0.077* | -0.093** | -0.091*** | -0.901*** | 0.368*** | -0.144*** | -0.294*** | -0.160*** | -0.035** | -0.291*** | -0.165*** | -0.040 | 1 |  |  |  |  |
| Change PGA 1^st^ 6 mnths | 0.036 | 0.032 | -0.059 | -0.178*** | -0.666*** | 0.092 | -0.124* | -0.079 | -0.103 | -0.062 | -0.022 | -0.041 | 0.322*** | 1 |  |  |  |
| Change PGE 1^st^ 6 mnths, | 0.020 | 0.028 | -0.021 | 0.032 | 0.061 | -0.595*** | -0.254*** | -0.330*** | -0.155** | 0.054 | 0.036 | 0.040 | 0.048 | 0.177*** | 1 |  |  |
| Change CHAQ 1^st^ year | 0.042 | -0.011 | 0.050 | -0.182*** | -0.182*** | -0.280*** | -0.589*** | -0.323*** | -0.195*** | -0.146*** | -0.072 | 0.026 | 0.181*** | 0.283*** | 0.116* | 1 |  |
| Change pain 1^st^ year | 0.041 | 0.079 | 0.043 | -0.131*** | -0.149*** | -0.366*** | -0.320*** | 0.576*** | -0.176*** | -0.097* | -0.037 | -0.050 | 0.098 | 0.330*** | 0.074 | 0.557*** | 1 |
| All values are pearson correlations adjusted from 50 multiply imputed datasets. PGA, Physician’s global assessment; PGE, Parent’s global evaluation of wellbeing; CHAQ, Childhood Health Assessment Questionnaire; AJC, Active Joint Count; MFQ, Moods and feelings questionnaire; DMARDS, Disease modifying antirheumatic drugs. *p value≤0.05, from adjusted multiply imputed Pearson correlations. **p value≤0.005, from adjusted multiply imputed Pearson correlations. ***p value≤0.0005, from adjusted multiply imputed Pearson correlations. | | | | | | | | | | | | | | | | | |
